# Robust Muscle Activity Onset Detection Using an Unsupervised Electromyogram Learning Framework

**DOI:** 10.1371/journal.pone.0127990

**Published:** 2015-06-03

**Authors:** Jie Liu, Dongwen Ying, William Z. Rymer, Ping Zhou

**Affiliations:** 1 Sensory Motor Performance Program, Rehabilitation Institute of Chicago, Chicago, United States of America; 2 Institute of Acoustics, Chinese Academy of Sciences, Beijing, China; 3 Department of Physical Medicine & Rehabilitation, Northwestern University, Chicago, United States of America; 4 Department of Physical Medicine & Rehabilitation, University of Texas Health Science Center at Houston; and TIRR Memorial Hermann Research Center, Houston, United States of America; 5 Biomedical Engineering Program, University of Science and Technology of China, Hefei, China; Duke University, UNITED STATES

## Abstract

Accurate muscle activity onset detection is an essential prerequisite for many applications of surface electromyogram (EMG). This study presents an unsupervised EMG learning framework based on a sequential Gaussian mixture model (GMM) to detect muscle activity onsets. The distribution of the logarithmic power of EMG signal was characterized by a two-component GMM in each frequency band, in which the two components respectively correspond to the posterior distribution of EMG burst and non-burst logarithmic powers. The parameter set of the GMM was sequentially estimated based on maximum likelihood, subject to constraints derived from the relationship between EMG burst and non-burst distributions. An optimal threshold for EMG burst/non-burst classification was determined using the GMM at each frequency band, and the final decision was obtained by a voting procedure. The proposed novel framework was applied to simulated and experimental surface EMG signals for muscle activity onset detection. Compared with conventional approaches, it demonstrated robust performance for low and changing signal to noise ratios in a dynamic environment. The framework is applicable for real-time implementation, and does not require the assumption of non EMG burst in the initial stage. Such features facilitate its practical application.

## Introduction

The function of muscle activity onset detector using surface electromyogram (EMG) is to distinguish occurrence of active muscle activity from baseline. Accurate muscle activity onset detection is one of the fundamental tasks in many applications of surface EMG such as posture or gait analysis, myoelectric control of prosthetic or orthotic devices. Various techniques for the determination of onset time of muscle activity have been proposed, in which the majority of utilized parameters are associated with EMG signal amplitude, such as the envelope, the average rectified value and the root mean square value of EMG time series [1–5]. One limitation of amplitude based parameters is that they are very sensitive to background noise level changes. The performance of these amplitude-based methods degrades as the signal to noise ratio (SNR) of the processed signal decreases. To overcome this difficulty, several methods such as double threshold detector [6,7], wavelet template matching [8,9], statistical criterion determination [10–14], Teager-Kaiser energy (TKE) operator conditioning [15–17], and sample entropy analysis [18] have been proposed to improve the performance of muscle activity onset detection, particularly when the SNR of the surface EMG is low.

While most of the previous methods focused on improvement of performance for muscle activity onset detection at relatively low SNRs, there are more challenges we need to address to achieve robust onset detection performance. For example, in a dynamic environment the SNR may not remain constant, which imposes a difficulty in maintaining good muscle activity onset detection performance. Moreover, with most of the previous methods, good performance is possible only when *a priori* knowledge of the processed signal is known or correctly estimated. Usually, the noise characteristics can be estimated from baseline recordings and this information is used to help differentiate the EMG activity from baseline noise. Such estimation process used for determination of threshold can be viewed as a supervised learning process. How to perform muscle activity onset detection using an unsupervised learning process needs further investigation. Finally, for some applications such as myoelectric prosthesis control, the design for onset detection should be applicable for a real-time implementation.

In this study, we propose a novel muscle activity onset detection technique based on an unsupervised EMG learning framework, which can be adaptive to a dynamic environment and also applicable to real-time detection. The unsupervised learning framework was developed based on a sequential Gaussian mixture model (GMM). It utilizes the energy distribution in Mel-spaced frequency bands of the signal as its feature parameter. The GMM consists of two Gaussian distributions, modeling either noise or surface EMG signals. Such machine learning based techniques have recently demonstrated their superiority in discriminating speech signal from background noise for voice activity detection [19–21]. In this study, the unsupervised learning based on a sequential GMM was applied to both simulated and experimental surface EMG signals to evaluate its performance for muscle activity onset detection. We show that the sequential GMM can identify bursts of surface EMG and is robust to low and changing SNRs. Furthermore, the algorithm has a potential for online detection and does not require the assumption of non EMG burst in the initial stage. These are important features for its practical application.

The rest of the paper is organized as follows. Section 2 of the paper introduces the proposed statistical framework and its implementation for muscle activity onset detection. In Section 3, a performance evaluation was conducted by comparing the proposed muscle activity onset detection method with several previously developed approaches, followed by a conclusive discussion in Section 4.

## Theoretical Background

The muscle activity onset detection method developed in this study is to model the subband logarithmic energy of EMG signal using an unsupervised learning framework based on GMM. In brief, for each subband of the processed signal, the logarithmic value of the absolute magnitude sum was calculated, and this logarithmic energy was smoothed to form an envelope for classification. The posterior distributions of the logarithmic energy for EMG burst and non-burst were characterized by two Gaussian models. The parameters of the two-component GMM were estimated using an unsupervised approach. The EMG burst/non-burst classification was performed at each of the subbands as described in the following subsections, and the final decision was obtained by a voting procedure.

### Modeling log-power sequences with GMM

**Fig 1:**
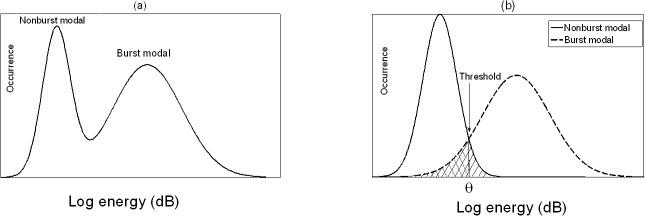
Schematic illustration of logarithmic energy distribution of a frequency band. (a) Distribution of EMG signal contaminated by noise. (b) Distributions of EMG burst and non-burst. The shadow denotes the classification error.

The unsupervised framework is implemented by using the model-based clustering [22], where the log-power sequence is modeled by a two-component GMM. A GMM considers a log-power sequence in a causal window of *L* samples, $x_{\ell }≜\{x_{\ell -L+1},\ldots ,x_{\ell }\}$. The EMG burst and non-burst logarithmic powers are assumed to follow a Gaussian distribution, respectively. The log-power distribution of observed signal is modeled by a GMM (Fig 1A), in which the probability of each component is represented by a Gaussian model (Fig 1B). In the following sections, $\lambda_{\ell }$ denotes the parameter set of GMM that is estimated from log-power sequence $x_{\ell }$ (see below for details). Let $s_{\ell }=1$ and $s_{\ell }=0$ respectively indicate the two hypotheses of EMG presence and absence in the ℓth frame. The GMM probability density function is given by

$$p(x_{\ell }|\lambda_{\ell })=\sum_{s_{\ell }}p(x_{\ell },s_{\ell }|\lambda_{\ell })=\sum_{s_{\ell }}p(s_{\ell }|\lambda_{\ell })p(x_{\ell }|s_{\ell },\lambda_{\ell }),$$ (1)

where $p(s_{\ell }|\lambda_{\ell })$ represents the *a priori* distribution of EMG burst presence/absence, and $p(x_{\ell }|s_{\ell },\lambda_{\ell })$ is the conditional probability density function of EMG presence/absence, given by

$$p(x_{\ell }|s_{\ell }=i,\lambda_{\ell })=\frac{1}{\sqrt{2\pi \kappa_{i,\ell }}}\text{exp}\{-\frac{1}{2}{\left(x_{\ell }-\mu_{i,\ell }\right)}^{2}/\kappa_{i,\ell }\},$$ (2)

where $\mu_{i,\ell }$ and $\kappa_{i,\ell }$ are the mean and variance of the Gaussian distribution for the given hypothesis $s_{\ell }=i$, respectively (*i* = 0 or 1).

The modeling problem involves estimating the parameter set $\lambda_{\ell }=\{w_{\ell },\mu_{\ell },\kappa_{\ell }\}$, where $\mu_{\ell }≜\{\mu_{0,\ell },\mu_{1,\ell }\}$, $\kappa_{\ell }≜\{\kappa_{0,\ell },\kappa_{1,\ell }\}$, and $w_{\ell }≜\{w_{0,\ell },w_{1,\ell }\}$. In theory, the prior information about the parameters is helpful to improve the parameter estimation. However, the priors depend on the background noise in this study. It is difficult to pre-train the priors that can cover all the noisy situations. Therefore, the priors on the parameters are ignored in the maximum likelihood (ML) estimation. Given a log-power training sequence $x_{\ell }$, a ML estimation of parameter set $\lambda_{\ell }$ is given by

$$\lambda_{\ell }=\arg\;\max_{\lambda }\;\sum_{s_{\ell }}p(x_{\ell },s_{\ell }|\lambda )$$ (3)

From the two distributions, an optimal threshold $\theta_{\ell }$ satisfying Eq 4 can be derived to minimize the classification error (Fig 1B).

$$p(\theta_{\ell }|s_{\ell }=1,\lambda_{\ell })p(s_{\ell }=1|\lambda_{l})=p(\theta_{\ell }|s_{\ell }=0,\lambda_{\ell })p(s_{\ell }=0|\lambda_{l}),$$ (4)

where $p(s_{\ell }=1|\lambda_{l})$ and $p(s_{\ell }=0|\lambda_{l})$ are the *a priori* probabilities of burst/non-burst, respectively. The *a prior* probabilities are equivalent to the weight coefficients $w_{\ell }$., i.e $w_{\ell }=p(s_{\ell }|\lambda )$. The samples with logarithmic energy less than $\theta_{\ell }$ are determined as background noise (non-burst), while greater than $\theta_{\ell }$ are determined as EMG burst.

### Sequential estimation of GMM parameters

The basic batch processing algorithm for estimating GMM parameters is the expectation–maximization (EM) algorithm [23]. Reconstruction of GMM at each period results in a heavy computational load and consumption of extra memory. To promote computation efficiency, a sequential method can be used to adapt GMM parameters. The current work presents a first-order sequential scheme, where new model $\lambda_{\ell +1}$ is a function of new observation $x_{\ell +1}$ and previous model $\lambda_{\ell }$. The model is sequentially updated frame by frame after constructing the initial model using the first *M* (M≤ℓ) frames through the EM algorithm. The sequential scheme is described below. For the sequence $x_{\ell }$ ($c_{\ell }$ is the index of the first sample),

$$w_{i,\ell }=\frac{1}{L}\sum_{t=c_{\ell }}^{\ell }p(s_{t}=i|x_{t},\lambda_{\ell }),$$ (5)

$$\mu_{i,\ell }=\frac{\sum_{t=c_{\ell }}^{\ell }x_{t}p(s_{t}=i|x_{t},\lambda_{\ell })}{Lw_{i,\ell }},$$ (6)

$$\kappa_{i,\ell }=\frac{\sum_{t=c_{\ell }}^{L}{\left(x_{t}-\mu_{i,\ell }\right)}^{2}p(s_{t}=i|x_{t},\lambda_{\ell })}{Lw_{i,\ell }},$$ (7)

where

$$p(s_{t}=i|x_{t},\lambda_{\ell })=\frac{w_{i,\ell }p(x_{t}|s_{t}=i,\lambda_{\ell })}{\sum_{s_{t}}w_{i,\ell }p(x_{t}|s_{t}=i,\lambda_{\ell })}$$ (8)

This is a high-order regressive process. We simplify this process into a first-order regressive process, and enable new model $\lambda_{\ell +1}$ to be a function of old model $\lambda_{\ell }$ and new observation $x_{\ell +1}$. Suppose that the GMM varies with time slowly, $\lambda_{\ell }\approx \lambda_{\ell -1}$ at time ℓ. Accordingly we have the relationship, $\sum_{t=c_{\ell }}^{\ell }p(s_{t}=i|x_{t},\lambda_{\ell })\approx \sum_{t=c_{\ell }}^{\ell }p(s_{t}=i|x_{t},\lambda_{\ell -1})$. The summation is approximated by the zero-order moment, $\sum_{t}p(s_{t}=i|x_{t},\lambda_{\ell -1})\approx Lw_{i,\ell }$, according to Eq 5. Combining these relationships, we have the following equation:

$$\sum_{t=c_{\ell }+1}^{\ell }p(s_{t}=i|x_{t},\lambda_{\ell })\approx (L-1)w_{i,\ell }$$ (9)

Substituting Eq 9 into Eq 5, we obtain

$$w_{i,\ell +1}=\frac{\left(L-1)w_{i,\ell }+p(s_{\ell +1}=i|x_{\ell +1},\lambda_{\ell }\right)}{L}$$ (10)

Let *α* = (*L* − 1)/*L*, we obtain the iterative equation

$$w_{i,\ell +1}=\alpha w_{i,\ell }+(1-\alpha )p(s_{\ell +1}=i|x_{\ell +1},\lambda_{\ell }),$$ (11)

where *α* (0 < *α* ≤ 1) denotes a forgetting factor; the conditional probability $p(s_{\ell +1}=i|x_{\ell +1},\lambda_{\ell })$ is calculated via Eq 8.

With the same principle, the summation item in Eq 6 can be approximated by the 1st-order moment

$$\sum_{t=c_{\ell }+1}^{\ell }p(s_{t}=i|x_{t},\lambda_{\ell })x_{t}\approx (L-1)w_{i,\ell }\mu_{i,\ell }$$ (12)

Substituting Eq 12 into Eq 6, we obtain

$$\mu_{i,\ell +1}=\frac{\alpha w_{i,\ell }\mu_{i,\ell }+(1-\alpha )p(s_{\ell +1}=i|x_{\ell +1},\lambda_{\ell })x_{\ell +1}}{w_{i,\ell +1}}$$ (13)

Accordingly, the summation item in Eq 7 can be approximated by the 2nd-order moment

$$\sum_{t=c_{\ell }+1}^{\ell }p(s_{t}=i|x_{t},\lambda_{\ell }){\left(x_{t}-\mu_{i,\ell +1}\right)}^{2}\approx (L-1)w_{i,\ell }\kappa_{i,\ell }$$ (14)

Substituting Eq 14 into Eq 7, we obtain

$$\kappa_{i,\ell +1}=\frac{\alpha w_{i,\ell }\kappa_{i,\ell }+(1-\alpha )p(s_{\ell +1}=i|x_{\ell +1},\lambda_{\ell }){\left(x_{\ell +1}-\mu_{i,\ell +1}\right)}^{2}}{w_{i,\ell +1}}$$ (15)

Thus, $\lambda_{\ell +1}$ can be derived from $\lambda_{\ell }$ and $x_{\ell +1}$ using the above sequential scheme (Eqs 8, 11, 13, and 15). Then, the time-varying threshold $\theta_{\ell +1}$ can be determined from Eq 4 which decides whether $x_{\ell +1}$ is identified as EMG burst or non-burst.

### Constraints on GMM

The binary-component GMM works well when both EMG burst and non-burst signals are present in a band. In the absence of EMG burst, the burst component is difficult to model. To deal with this situation, constraints of the GMM are derived from the relationships between EMG burst and non-burst distributions. Considering that non-burst represents background noise whereas EMG burst represents the superposition of noise and clean EMG burst signals, the non-burst mean $\mu_{0,\ell }$ is smaller than the EMG burst mean $\mu_{1,\ell }$. The mean difference $\mu_{1,\ell }-\mu_{0,\ell }$ represents the posterior SNR. In a similar relationship, the background noise is more stationary than EMG burst, thus the non-burst variance is smaller than the EMG burst variance. Such relationships are reflected in the binary-state GMM by two constraints.

$$\mu_{1,\ell }=\max\;\{\mu_{1,\ell },\mu_{0,\ell }+\delta \},$$ (16)

where *δ* > 0, and

$$\kappa_{1,\ell }=\max\;\{\kappa_{0,\ell },\kappa_{1,\ell }\}.$$ (17)

These two constraints can solve the potential problems in the absence of EMG burst while they have insignificant effects on the GMM parameters when EMG burst is present. Without these constraints, when the binary-state GMM is continuously updated by noise powers, the mean of EMG burst decreases and gradually approaches the mean of non-burst. GMM eventually loses its capability to discriminate burst/non-burst components. The constraints enforce the burst mean to be at least *δ* dB larger than the non-burst mean. So the discrimination capability can be maintained in the absence of EMG burst. Under such circumstances, the burst component is transformed into a virtual component with mean $\mu_{0,\ell }+\delta$ and variance $\kappa_{0,\ell }$. *δ* is an important parameter that makes a tradeoff between misdetection of weak burst spectral components and false alarm of strong non-burst spectral components. A large *δ* is helpful to reduce the false alarm while a small *δ* is helpful to reduce the misdetection.

It should be noted that in the sequential process, $w_{1,\ell }$ will approach 0 if the model is continuously updated by non-burst signals. As a result, it is difficult to transition from non-burst to burst states. For this reason, another constraint is set for $w_{1,\ell }$:

$$\begin{array}{l} w_{1,\ell }=\max\;\{w_{1,\ell },\epsilon \},\\ w_{0,\ell }=1-w_{1,\ell }, \end{array}$$ (18)

where *ϵ* is slightly greater than zero. Both Bayesian estimation [24] and ML estimation [25] can be used. It is noted that the prior information about the constraints can be incorporated into a Bayesian framework. However, it is difficult to derive a sequential scheme based on Bayesian framework with high computational efficiency. For simplicity, we propose the framework that consists of ML-based sequential GMM and constraints.

### Framework implementation

**Fig 2:**
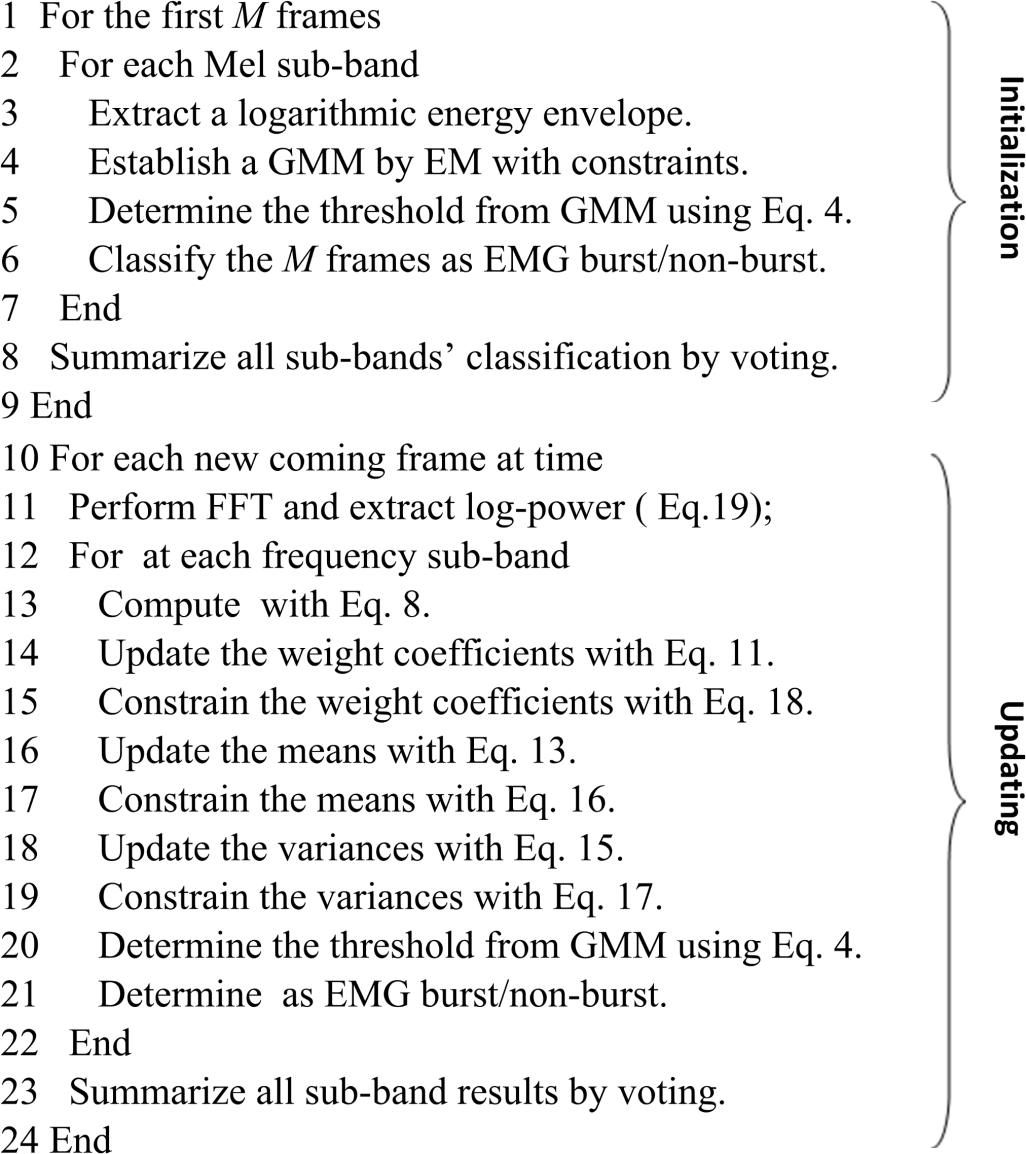
Process of EMG onset/offset detection using the sequential GMM.

The framework was implemented in MATLAB (v.7.12.0 105 R2011a, MathWorks Inc., Natick, MA). The sequential GMM runs on each subband in parallel. The principle of the optimal estimation of sequential GMM parameters lies in maximizing the likelihood, subject to the three constraints (Eqs 16, 17, 18) embedded into the sequential process. The initial model can be established using the EM algorithm. Fig 2 summarizes the EMG onset detection using the similar framework implementation as presented in [21].

In practical application, EMG burst may not be present during initialization, where only the noise component can be estimated from the observed data and the signal component is constructed by constraints. The minimum description length (MDL) selection principle [26] was utilized to determine whether the EMG burst was present in initialization. To calculate the logarithmic energy envelope of each subband, the EMG signal was divided into a series of overlapping analysis frames. It was then transformed into the frequency domain with discrete Fourier transform (DFT), and grouped into *N* Mel-scale subbands. For the *n*-th subband, its logarithmic energy is calculated as:

$${\bar{x}}_{\ell }=10\log_{10}\left[\frac{1}{f_{n+1}-f_{n}}\sum_{j=f_{n}}^{f_{n+1}-1}{\left|Y_{\ell ,j}\right|}^{2}\right]$$ (19)

where *f*_*n*_ is the frequency subband index corresponding to the *n*-th Mel scale, *n* = 0,1,…,*N*. $Y_{\ell ,j}$ is the *j*-th DFT coefficient of the ℓth frame. The energy sequence $\left\{{\bar{x}}_{\ell }|\ell =0,1,2...\right\}$ was finally smoothed using a five-point medium filter to form the envelope $\left\{x_{\ell }|\ell =0,1,2...\right\}$.

Specifically, the EMG signal was first chopped into a series of frames using a Hanning window (window length: 32 ms, overlapping step: 14 ms). The signal of each frame was subsequently transformed to the frequency domain by the fast Fourier transform (FFT) and grouped into eight Mel-scale subbands: 0–82.1 Hz, 82.1–173.8 Hz, 173.8–276.3 Hz, 276.3–390.9 Hz, 390.9–518.8 Hz, 518.8–661.8 Hz, 661.8–821.5 Hz, and 821.5 Hz -1 kHz, by using the logarithmic value of the absolute magnitude sum of included FFT bins. The FFT bins were partitioned into eight subbands with the following groups: (1–3), (4–6), (7–9), (10–13), (14–17), (18–22), (23–27), (28–33).

The two cluster means were used corresponding to the baseline and the EMG burst, respectively. The mixture of all the sub-band results was processed by a voting procedure. There was an adaptive threshold at each band. The number of votes was the amount of frequency bands with logarithmic power greater than the adaptive threshold. The number of votes ranged from 0 to 8.

## Performance Evaluation

### Surface EMG simulation

To evaluate the performance of the unsupervised learning framework for detection of muscle activation, a series of surface EMG signals were simulated by filtering white Gaussian noise with a shaping filter modeling the characteristics of typical surface EMG [27]. The shaping filter has the form [28]

$$H_{sf}(s)=\frac{ks{\left(2\pi f_{h}\right)}^{2}}{(s+2\pi f_{l}){\left(s+2\pi f_{h}\right)}^{2}}$$ (20)

where *s* is the Laplace variable, *k* is a scaling factor. The band-pass filter cut-off frequencies *f*_*l*_ and *f*_*h*_ were set to 80 Hz and 120 Hz, respectively.

To simulate experimental surface EMG with different noise levels, an independently generated zero-mean white Gaussian noise was added to simulated clean EMG signals. The standard deviation of the noise was determined in such a way that the noise level resulted in different SNRs (20, 15, 10, 8, 5, 4, 3, and 2 dB, respectively) of simulated surface EMG signals. For each SNR, 60 trials of the noise were added to 60 trials of the simulated clean EMG, generating 60 testing signals. Based on these signals, time-varying surface EMG recordings can also be simulated. All the simulated signals were sampled at 2 kHz and processed with a 6th order Butterworth band-pass filter at 20–500 Hz.

### Different methods used for performance comparison

The performance of the unsupervised learning framework was compared with three previous methods for muscle activity onset detection. The first method is based on amplitude thresholding of rectified surface EMG signals (denoted as AMP-based method). A threshold was set to be three standard deviations of the background noise of the surface EMG signal, as suggested in previous studies [5]. The second method applies the Teager-Kaiser energy (TKE) operation on raw surface EMG signals. The threshold was set to be six times the standard deviation of the TKE domain baseline as suggested by [16]. The third method is the double threshold algorithm developed by Bonato et al. [6]. For the unsupervised framework, parameters used for implementing the sequential GMM algorithm included *α* = 0.99, *δ* = 3.5, *ε* = 0.03. The number of frames used to initialize the GMM was 80 (*M* = 80). The frame length was 32 ms, and the frame shift was 14 ms. The number of subbands was 8 (*N* = 8). Among different parameters, *δ* and *M* used for initialization are the most important. *δ* influences the false alarm and the misdetection rate. A relatively small *M* reduces processing delay and facilitates real time implementation whereas to guarantee the non-EMG burst presence during the initialization, it is necessary to set a sufficiently large *M* (to bridge adjacent EMG burst segments). In general, setting *M* = 80 can achieve a good tradeoff. The onset performance was evaluated by the latency *τ*, defined as the absolute difference between the true onset time *t*_0_ and the detected onset time *t*_*d*_:

$$\tau =|t_{d}-t_{0}|$$ (21)

### Performance evaluation with simulated signals

**Fig 3:**
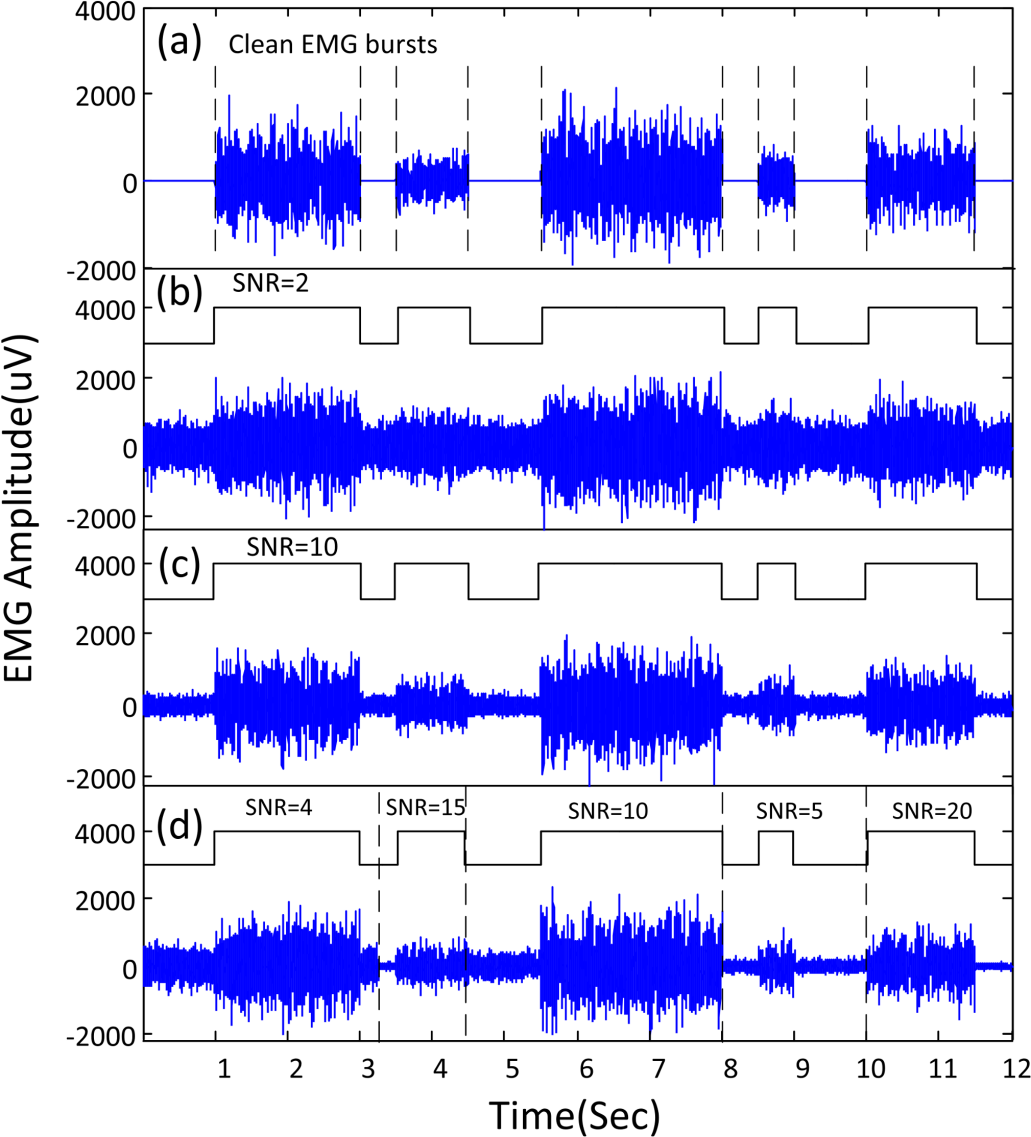
Simulated EMG bursts and the corresponding detection performance of the unsupervised learning framework. The detected segments with muscle activity were highlighted by the rectangular envelope built on the basis of the onset/offset estimates provided by the unsupervised learning framework. (a) Simulated clean EMG trial without added noise, which is composed of five EMG bursts with different durations and amplitudes. The corresponding actual onsets and offsets are marked by vertical dashed lines. (b) Simulated EMG signals at SNR level of 2 dB and the muscle activity onset detection performance. (c) Simulated EMG signals at SNR level of 10 dB and the muscle activity onset detection performance. (d) Simulated EMG signals with time-varying SNR levels and the muscle activity onset detection performance (the dashed lines indicate different signal segments for calculating SNRs).

Fig 3 shows an example of simulated EMG trials with different SNRs. The trial is composed of five EMG bursts with different durations and levels of EMG power (or SNRs). The sequential GMM method was able to adapt to the different (Fig 3B and 3C) or varying (Fig 3D) SNRs of the surface EMG signal for reliable muscle activity onset detection.

**Fig 4:**
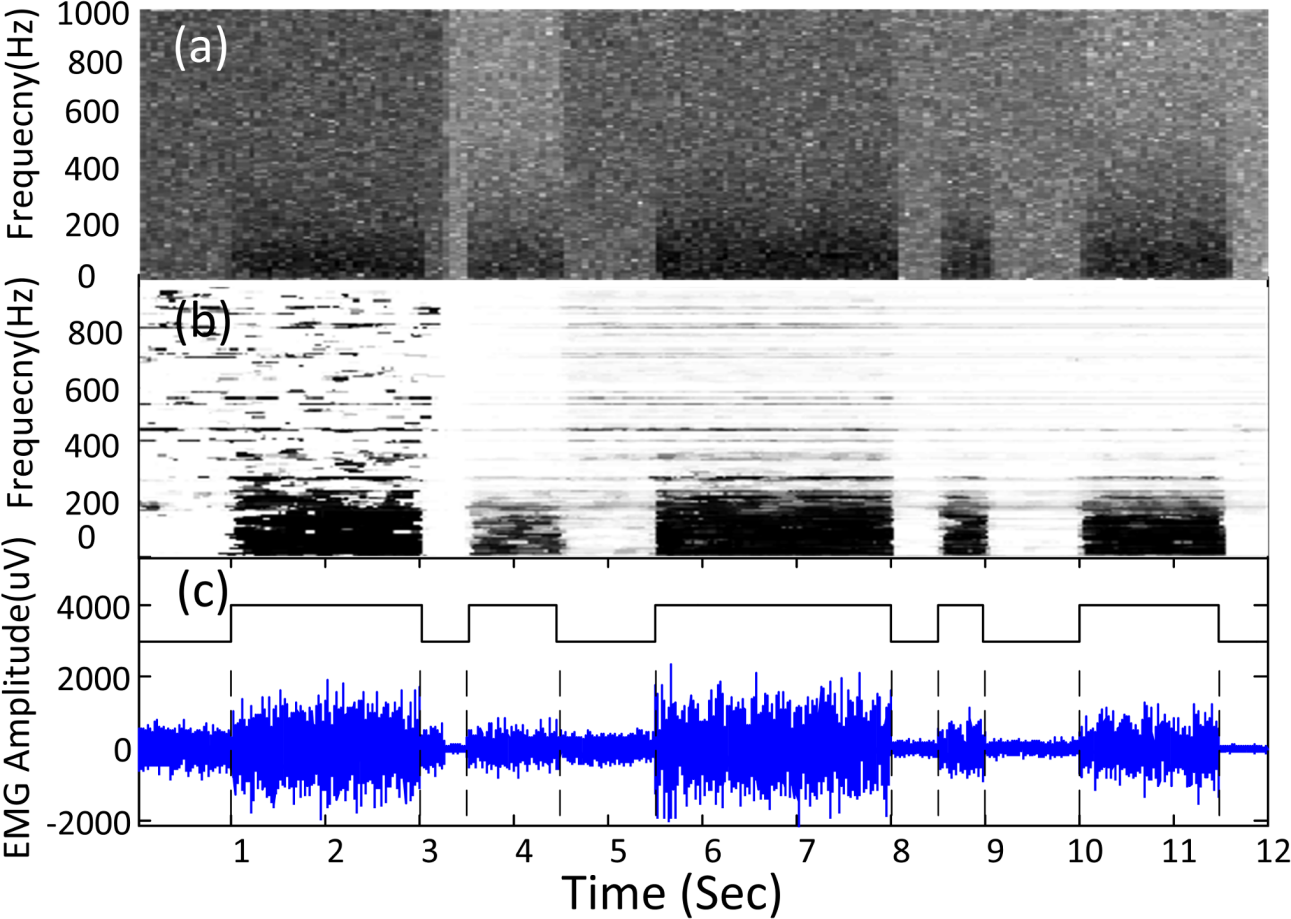
EMG burst presence probability in the time-frequency domain estimated by the unsupervised learning framework. (a) Magnitude spectrogram of the simulated EMG signal with time-varying SNR levels. (b) Spectrogram of EMG burst presence probability. The transition from white to black corresponds to probability changing from 0 to 1. (c) Simulated EMG signals with time-varying SNR levels and the muscle activity onset detection performance (the rectangular envelope); the corresponding actual onsets and offsets are marked by vertical dashed lines.

In addition to detecting frames with muscle activity, the sequential GMM method can estimate the presence probability of the EMG burst in the time-frequency domain. One example is shown in Fig 4, where the spectrograms of the EMG signal (Fig 4A) and the EMG burst presence probability (Fig 4B) are presented for an EMG trial corrupted by white noise with different SNR values (Fig 4C). At each subband, the presence probability sequence describes the EMG burst activity in a soft manner. The transition from white to black in Fig 4 corresponds to probability changing from 0 to 1. It is noted that compared with the EMG spectrogram, the EMG burst presence probability demonstrates a consistent but discriminative pattern to detect presence of EMG burst from background noise. Thus the EMG burst spectral structure can be more clearly described by the time–frequency EMG burst presence probability. Note that the presence of EMG burst can be determined by comparing the votes with an empirical voting threshold. If the votes are higher than the threshold, the EMG burst is assumed to be present. Surface EMG burst is more prevalent in relatively low frequencies. For this reason, more subbands can be set in low than high frequencies to facilitate detection of the burst.

**Fig 5:**
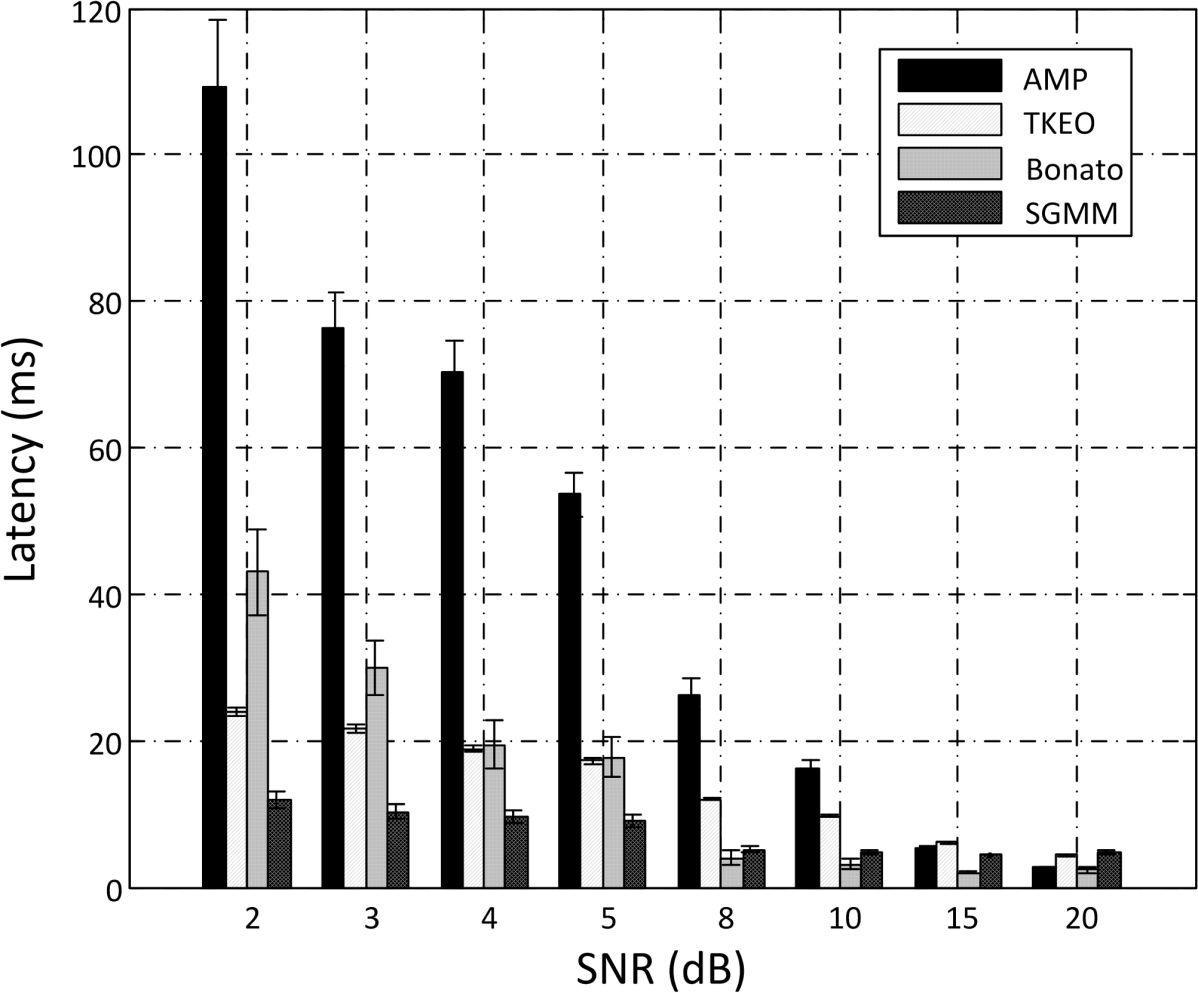
Comparison of onset detection performance using different methods (mean ± standard error). AMP: the conventional amplitude thresholding method; TKEO: the method based on TKE operation conditioning; Bonato: the double threshold algorithm developed by Bonato et al. [6]; SGMM: the sequential GMM based unsupervised learning method. For each SNR level, the mean latency was averaged over 60 trials of simulated surface EMG signals.

Fig 5 summarizes the muscle activity onset detection performance of the four different methods. Note that the simulated EMG with only one EMG burst was used for the comparison of different onset detection methods. The precise onset time for all the testing signals was known as 1 s over the entire 4 s recording period. It was observed that as the SNR of the signal decreased from 20 to 2 dB, the latency of the onset detection for all the methods tended to increase. For each signal condition, a significant difference was observed between different methods (repeated measures ANOVA, p < 0.05). Among all the methods, the unsupervised learning framework based on the sequential GMM achieved the best performance at relatively low SNR levels (for SNRs lower than 8 dB). Moreover, it demonstrated the most robust performance with varying SNR levels.

### Testing on experimental EMG signals

**Fig 6:**
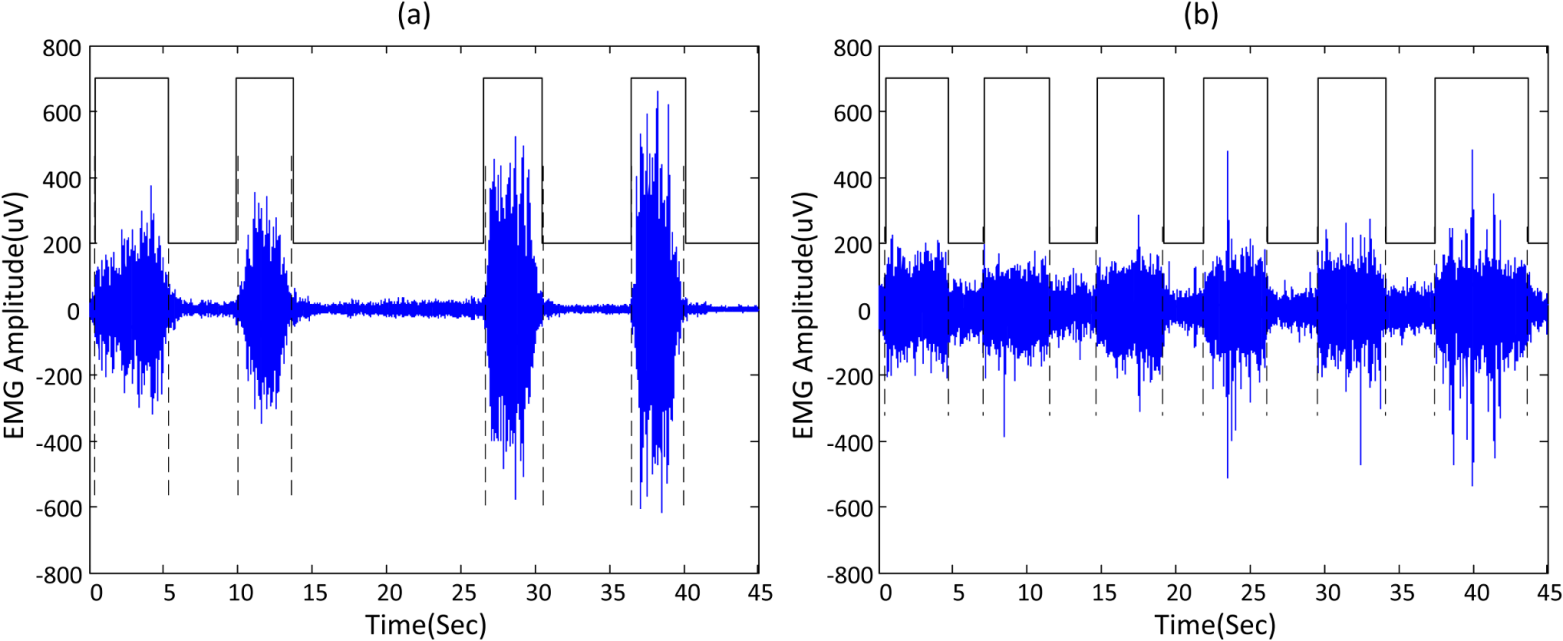
Experimental surface EMG onset detection. Examples of the experimental surface EMG signals with relatively (a) high and (b) low SNRs, and the muscle activity segments identified by the unsupervised learning framework.

Fig 6 demonstrates examples of applying the proposed method to experimental surface EMG signals for muscle activity onset detection. The signals were sampled at 2 kHz from paretic forearm muscles of two cerebral palsy (CP) subjects with a band-pass filter setting at 10–500 Hz. The objective of such recordings with the CP subjects was to evaluate the neural control information in their affected muscles using advanced myoelectric pattern recognition techniques [29]. The study was approved by the Institutional Review Board of Northwestern University (Chicago, USA) (Reference number: STU00023682). All the participants provided their written informed consent to participate in this study. Fig 6 shows two 45 s time periods during which each subject performed repetitions of voluntary muscle contraction. The detected segments with muscle activity were highlighted by the rectangular envelope built on the basis of the onset/offset estimates provided by the unsupervised learning framework. It can be observed that the developed framework was able to detect muscle activity at different SNR levels. Note that if the muscle activity occurred at the initial state, the framework can still correctly detect the muscle activity onset without the assumption of non-burst beginning.

Using visual inspection of the experimental signals as a standard, the muscle activity onset detection performance of the four different methods was further examined in a more quantitative way. Averaged from 10 trials of experimental surface EMG signals of 5 CP subjects, the unsupervised learning framework based on the sequential GMM achieved the detection latency of 35.85 ± 5.31 ms (mean ± standard error), whereas all the other three methods produced longer latencies (AMP: 122.15 ± 67.98 ms; TKEO: 107.20 ± 58.00; the double threshold method: 51.95 ± 15.80 ms). Note that the threshold setting for all the methods was the same as used for simulated signals.

## Discussion

This study develops a statistical framework based on unsupervised learning to model the EMG burst and non-burst distributions in the frequency domain for muscle activity onset detection. A sequential GMM was employed to discriminate between burst and non-burst distributions at each Mel-spaced frequency band, using the energy distribution as feature parameter. The Mel-frequency warping method as used in this study emphasizes low frequency information of EMG signals. Compared with those based on equal-partition bands, the method based on Mel-frequency bands has higher resolution in lower frequencies [30]. Mel-frequency cepstral coefficients were employed in previous studies for developing EMG-based speech recognition systems [31, 32]. In the current application, GMM concerns not only the posterior distribution (two Gaussian models), but also the *a priori* distribution (weight coefficients). The distribution is described by an unsupervised learning framework including the posterior probability, *a priori* probability, and constraints to GMM. Specifically, the *a priori* probabilities are equivalent to the weight coefficients of GMM, which are sequentially estimated. Other EMG features that satisfy the bimodal distribution can also be applied to this unsupervised framework and have potential to further improve the muscle activity onset detection performance.

The unsupervised learning framework used in this study considers *a priori* distributions of EMG burst and non-burst signals, as well as the distribution relationships between the signals. Compared with conventional statistical models, it has advantages in both the initialization process and the sequential process. At initialization, both the EMG burst and non-burst models are simultaneously constructed based on the criterion of maximum likelihood. The proposed model can be correctly initialized regardless of the presence or absence of EMG burst in the beginning of the process. In the updating process, the statistical models are updated in a soft manner, controlled by the presence probability of the EMG burst. The sequential GMM based muscle activity onset detection not only provides EMG presence information in the frame level, but also more detailed EMG presence probability in each frequency component. The non-burst information in the EMG frames can be employed to update models.

Because of the above advantages, compared with previously developed methods, the novel muscle activity onset detection method developed in this study is characterized by several features. Firstly, the method not only works well in low SNRs, but also is adaptive to changing SNRs, thus being capable of robust muscle activity onset detection in a dynamic environment. Secondly, the proposed algorithm is able to run in an online manner, so the muscle activity onset detection can be applied to real-time systems. Finally, the method can correctly estimate the muscle activity onset without the assumption of non EMG burst in the initial state. Thus, this muscle activity onset detection scheme is more practical than conventional ones.

The proposed unsupervised learning method based on sequential GMM was applied to simulated and experimental surface EMG signals to evaluate its performance for muscle activity onset detection. However, one limitation of the current study is that the proposed method was only tested using surface EMG data from a small number of CP subjects. We acknowledge that it is important to perform extensive testing of the novel method with a wide group of patients with different neuromuscular diseases or other disabilities (such as amputee subjects). Thus it remains our future work to further validate the clinical usability of the proposed method for muscle activity onset detection.
